# Oxidative stress-mediated hepatotoxicity in rats induced by ethanol extracts of different parts of *Chloranthus serratu*s

**DOI:** 10.1080/13880209.2020.1859552

**Published:** 2020-12-23

**Authors:** Shuping Sun, Yang Wang, Yunyan Du, Qi Sun, Lijuan He, Enze Zhu, Jiarong Li

**Affiliations:** a College of Pharmacy, Wannan Medical College, Wuhu, Anhui, China; b Institute of Natural Daily Chemistry, Wannan Medical College, Wuhu, Anhui, China; c College of Pharmacy, Heilongjiang University Of Chinese Medicine, Harbin, Heilongjiang, China

**Keywords:** Chloranthaceae, liver damage, pathological changes, oxidative damage, toxic mechanism, Nrf2/HO-1 pathway

## Abstract

**Context:**

*Chloranthus serratus* (Thunb.) Roem. et Schult. (Chloranthaceae) is an herb widely used as a folk medicine treating inflammatory diseases, although it is toxic.

**Objective:**

To investigate hepatotoxicity and related mechanisms induced by ethanol extracts of different parts of *C. serratus* in rats.

**Materials and methods:**

Sprague Dawley rats were divided into control (Con), ethanol extract of roots (ER), stems (ES), and leaves (EL) groups, and acute oral toxicity studies were conducted. The rats received doses of 4.14, 3.20, and 1.16 g/kg/d extracts for 14 days, respectively. Liver index, liver function and oxidative stress biomarkers, liver pathology, ultrastructure, TNF-α, ICAM-1, and Nrf2/HO-1 proteins expression levels were determined.

**Results:**

The LD_50_ of ER, ES, and EL were higher than 10.35, 8.05, and 2.90 g/kg/p.o., respectively. The liver indexes in the extract groups increased significantly. EL dramatically increased TP, GLB, AST, ALT, ALP, TBA, MDA, ICAM-1, and TNF-α levels (*p* < 0.01), and induced the most obvious pathological and ultrastructural changes. ES and EL obviously decreased the T-SOD, GSH, CAT, and CHOL levels. Nrf2 and HO-1 proteins expression was reduced significantly in ES (0.77 ± 0.06, 2.33 ± 0.20) and EL (0.23 ± 0.04, 2.14 ± 0.16) groups, and reduced slightly in ER (1.08 ± 0.10; 3.39 ± 0.21) group.

**Discussion and conclusion:**

ES and EL induce stronger hepatotoxicity than ER through oxidative stress and the Nrf2/HO-1 pathway, and the root is a better medicinal part, which provides a basis for clinical research, safe applications, and reasonable development of *C. serratus*.

## Introduction

The liver is not only an important metabolic organ but also the largest detoxifying organ in the human body, which plays an important role in sustaining life (Jeong et al. 2013). This organ is particularly vulnerable to long-term exposure to toxic substances. The poisons and wastes produced by the human body, as well as the poisons and drugs ingested into the body, rely on the liver for detoxification, thus causing damage to the liver. With the vigorous development of traditional Chinese medicine (TCM), drug-induced hepatotoxicity has gradually become an urgent issue in TCM research (Zargar 2014).

*Chloranthus serratus* (Thunb.) Roem. et Schult. (Chloranthaceae) is mainly distributed in Guangxi, Guizhou, Yunnan, and other provinces of China (Tag et al. 2010). According to the *Chinese Medicine Dictionary* (NUTCM 2006), *C. serratus* has many therapeutic effects, including dispelling wind, relieving pain, promoting blood circulation to dissipate blood stasis, and treating injuries from fall, rheumatic pain, and other symptoms. Moreover, the water-extractable components of *C. serratus* might exert an anti-inflammatory effect in lipopolysaccharide-stimulated macrophages (Sun et al. 2019b), and the anti-arthritic activity of different parts of *C. serratus* in adjuvant arthritis rats is related to inhibiting the release of inflammatory cytokines and improving antioxidant capacity (Sun et al. 2020b).

Despite its therapeutic effects, *C. serratus* has been shown to be toxic in clinical trials (NUTCM 2006). According to the *Chinese Medicine Toxicity Classification Standard* (Wu et al. 2007), it is considered as a slightly toxic TCM. *C. serratus* is rich in terpenoids, such as acolamone, chloranthalactone, shizukanolide, and zederone, and herbs containing triterpenoids have been confirmed to have hepatotoxicity (Chitturi and Farrell 2008; Zhang et al. 2013). When *C. serratus* is used to treat diseases, patients may be poisoned or even dead due to incorrect administration or misuse. The patients’ serum levels of aspartate aminotransferase (AST), alanine aminotransferase (ALT), and lactate dehydrogenase (LDH) were significantly increased (Zhou and Hu 1996). Autopsy results showed inflammatory cell infiltration and hepatic steatosis in patients with liver failure due to *C. serratus* intoxication (Zhang et al. 2010). In addition, dilatation of the hepatic sinusoid, congestion, and focal haemorrhage was observed in the liver tissues of mice treated with *C. serratus* extract (Zhang et al. 2006), indicating that *C. serratus* has hepatotoxicity.

The medicinal parts of *C. serratus* can be the whole grass, root, or arial part (Zhu et al. 2017). However, there have been no studies on the differences in hepatotoxicity of the different parts of *C. serratus*. The investigation shows that there are two methods of water decocting and wine immersion in folk and clinical applications. The commercially available prescription preparation ‘*Panax notoginseng* (Burk.) F.H.Chen, (Araliaceae) medicinal wine’ contains *C. serratus*. In this preparation, the extraction method of *C. serratus* belongs to the ethanol immersion method. In this study, the hepatotoxicity tests of the ethanol extracts of *C. serratus* roots, stems, and leaves were performed on rats by gavage to identify the part with the least toxicity. This study will not only improve our awareness of *C. serratus*, allowing us to take full advantage of its medicinal value, but also provide a basis for the prevention and treatment of *C. serratus* poisoning, as well as provide evidences for forensic identification, and lay the foundation for reasonable development and utilization of *C. serratus*.

## Materials and methods

### Experimental animals and ethics statement

Male Sprague-Dawley (SD) rats (200 ± 10 g, aged 6–8 weeks) were purchased from Nanjing Experimental Animal Centre, Nanjing, China (Certificate No. 18-014). The rats were housed at 23 ± 2 °C and 55 ± 5% RH for one week before the experiment. All experimental procedures used in this study were approved by the ethics committee of Wannan Medical College (WNMC No. 20180305).

### Reagents

Assay kits for malondialdehyde (MDA, cat: 20180613), total superoxide dismutase (T-SOD, cat: 20180616), glutathione (GSH, cat: 20180615), catalase (CAT, cat: 20180613), total protein (TP, cat: 20180610), alkaline phosphatase (ALP, cat: 20180609), AST (cat: 20180608), ALT (cat: 20180607), globulin (GLB, cat: 20180606), total bile acid (TBA, cat: 20180605), and cholesterol (CHOL, cat: 20180604) were purchased from Jiancheng Biotechnology Co., Ltd. (Nanjing, China). Mouse anti-β-actin (cat: 66240-1-lg), goat anti-mouse IgG (cat: BST12F21C50), rabbit anti-heme oxygenase-1 (HO-1, cat: ZP1039BP39), goat anti-rabbit IgG (cat: BST12L05A54), rabbit antitumor necrosis factor-α (TNF-α) (cat: BST12F21C48), mouse anti-intercellular cell adhesion molecule-1 (ICAM-1) (cat: BST12F21C50), and immediate SABC-POD (goat anti-rabbit IgG, cat: 12H25C) kit were purchased from Boster Biological Technology Co., Ltd. (Wuhan, China). Rabbit anti-nuclear factor erythroid-2 related factor 2 (Nrf2) (cat: 16396-1-AP) and rabbit anti-β-tubulin (cat: 16385-1-AP) antibodies were obtained from Proteintech Co., Ltd. (Chicago, IL, USA).

### Plant materials, extract preparation and fingerprint analysis

*C. serratus* was harvested from Yulin (Guangxi, China) in May 2018. With reference to the *Chinese Medicine Dictionary* (NUTCM 2006), it was confirmed to be authentic by Professor Jianhua Zhu (Wannan Medical College, Anhui, China). A voucher specimen of *C. serratus* (ANUB No. 14096, Xiaoping Zhang) was deposited at the Herbarium Center, Anhui Normal University, China. The entire plant was washed with tap water and dried naturally. The roots, stems, and leaves were separated and crushed into coarse powder, then stored at room temperature.

The coarse powder of *C. serratus* roots was immersed in 12-fold volume of 75% ethanol for 0.5 h, extracted for 1.5 h, and then extracted with 10-fold and 8-fold volumes of solvent for 1 h, respectively. The filtrate was pooled together for vacuum recovery and dried in a 45 °C vacuum oven (Yiheng Scientific Instrument Co., Ltd., Shanghai, China). Next, the sample was pulverized through an 80-mesh sieve to obtain the ethanol extract of the roots. The preparation methods of the stem and leaf ethanol extracts were the same as above. The ethanol extracts were stored in a desiccator at room temperature. The extraction rate (%) = weight of ethanol extract (g)/weight of coarse powder (g) × 100%, and the extraction rates of the roots, stems, and leaves were 15.35%, 11.9%, and 4.31%, respectively.

The sample solutions were obtained by dissolving 0.05 g of the ethanol extracts of roots (ER), stems (ES), and leaves (EL) in 75% ethanol to obtain a concentration of 1 mg/mL, respectively. After a blank correction with 75% ethanol, the solutions were scanned by using a Hitachi U-5100 spectrophotometer (Hitachi High-Tech Science Corporation, Japan) under the following spectral conditions: data mode: Abs; scanning range: 190–500 nm; scanning speed: 400 nm/min; delay: 0 s; response: fast; sampling interval: 1.0 nm; cycle time: 1.0 min; and slit width: 5.0 nm (Sun et al. 2019a, 2019b).

The sample solutions were obtained by accurately weighing 2 mg of the ER, ES, and EL, then dissolving with 500 μL of 50% methanol aqueous solution, respectively. Before HPLC separation using a ultra-high pressure liquid phase (Agilent 1260, Agilent Technologies), the samples were sonicated for 10 min at room temperature and centrifuged for 10 min, and then the supernatant fluid was filtered through 0.22 μm microporous membrane. In order to obtain the characteristic chromatograms data, blank correction was performed with 50% methanol aqueous solution. A C_18_ column (ZORBAX Eclipse XDB-C_18_, 250 mm × 4.6 mm, 5 μm) was used. The mobile phases were 0.2% phosphoric acid (A) and acetonitrile (B), and the elution gradient was 0–10 min, 95% A; 10–40 min, 80% A; 40–50 min, 30% A; 50–55 min, 5% A; 55–60 min, 95% A. The detection wavelength was 350 nm and the flow rate was 1.0 mL/min. The injection volume was 10 μL, and the column temperature was 40 °C.

### Acute oral toxicity study

Acute oral toxicity studies of the ER, ES, and EL were conducted. Each rat (4 rats/group) was orally administered a test dose of 10.35 g/kg ER, 8.05 g/kg ES, or 2.90 g/kg EL, respectively. If mortality was not observed, additional two rats were given the same dose of the extract. Then the gross morphological changes and mortality of these rats were observed for a period up to 48 h (Sun et al. 2020b). The purpose of acute toxicity test was to check the rationality of medication.

### Treatment of animals

Twenty-four male SD rats were randomly divided into four groups (6 rats/group): Control (Con), ER, ES, and EL groups. According to the interspecies equivalent dose conversion table (Wei et al. 2010) and pre-experimental results, the daily dose of rats (g/kg) = 3 g × 0.018 × 5 × extraction rate (ER = 15.35%; ES = 11.90%; EL = 4.31%) × 100 (multiple), where 3 g represents the mass of the roots, stems or leaves of *C. serratus*, i.e. the human daily dose; 0.018 represents the conversion factor; and 5 indicates that the dosage of 200 g per rat is converted into the dose per kilogram of body weight. The dosage for each group was 4.14, 3.20, and 1.16 g/kg/day, respectively. The rats in the extract groups were intragastrically administrated a mixture of extracts and 0.5% carboxymethylcellulose sodium (CMC-Na) solution. The rats in the Con group were administrated an equal volume of 0.5% CMC-Na solution once a day for 14 consecutive days. All the test animals were sacrificed on the 15th day.

### Observation of general conditions and weight changes

The rats were weighed on the 1st, 3rd, 7th, 11th, and 15th days to calculate the change rates of the body weight. The body weight on the first day was called the initial weight (IW), and the body weights on any other day were called the final weights (FWx, x = 3, 7, 11, and 15). The change rates of the body weights of the rats (%) = (FWx – IW)/IW × 100%. The vital signs of the rats were recorded, including activities, diet, fur appearance, urine, etc. On the 15th day, blood samples of the rats were collected from the medial retroorbital venous plexus with capillaries after anaesthetization by ether inhalation. The blood was centrifuged at 4 °C and 2500 rpm for 15 min. Then, the supernatant was collected and stored at −80 °C for subsequent experiments.

### Determination of the liver index

After blood collection, the rats still under anaesthesia were euthanized by cervical dislocation, then the livers were collected and weighed to calculate the liver index. Liver index (%) = weight of the liver (g)/weight of the rat (g) × 100%. The liver morphologies of the rats were observed and photographed.

### Histopathological analysis

The tissue samples were fixed in 10% formalin, embedded in paraffin, then sectioned and stained with haematoxylin and eosin (HE). The stained slices were examined with a CKX3 OLYMPUS Microscope (Kunshan Nopson Laboratory Supplies Technology Co., Ltd., Kunshan, China) by two experienced pathologists who were unaware of the animal treatment groups. The criteria for evaluating slices were based on the intensity and diffusion of vasodilatation and congestion, local necrosis, inflammatory cell infiltration, steatosis, edoema fluid, and eosinophilic changes. Finally, the counted values of each section were summed to determine the degree of degeneration. Scoring was performed as follows: 0–4 (no, low, moderate, high, and very high, respectively) (Yagmurca et al. 2007).

### Immunohistochemistry analysis

The paraffin slices were dewaxed, rehydrated with ethanol at gradually reduced concentrations, and incubated in sodium citrate buffer for antigen retrieval. After the slices were washed with phosphate-buffered-saline solution (PBS), they were treated with hydrogen peroxide to quench endogenous peroxidases, and then blocked with serum at 4 °C for 30 min. Subsequently, the slices were incubated with the anti-TNF-α and anti-ICAM-1 primary antibodies at 4 °C overnight and then incubated with the secondary antibodies at room temperature for 1 h. Afterwards, the slices were incubated with SABC reagent at 37 °C for 1 h, treated with DBA and re-stained with haematoxylin. After that, the slices were observed and photographed under a CKX3 OLYMPUS light microscope (Kunshan Nopson Laboratory Supplies Technology Co., Ltd., Kunshan, China). The percentage of positive expression was obtained by using ImageJ software and analysed quantitatively.

### Determination of liver function biomarkers

The serum was rewarmed at room temperature. The ALT, AST, TP, GLB, ALP, TBA, and CHOL contents were determined with an automatic biochemical analyser (Hitachi, Japan) according to the instructions of commercial assay kits.

### Assay of liver oxidative stress markers

The liver tissues were homogenized in an ice-water bath according to the ratio of the liver tissue mass (g):physiological saline (mL) = 1:9. After that, the homogenate was centrifuged at 4 °C and 2500 rpm for 20 min, and then the supernatant was collected to determine the MDA, T-SOD, GSH, and CAT contents according to the instructions of the kits.

### Ultrastructural examination

The tissue slices (1–2 µm) were fixed in 2.5% glutaraldehyde and then transferred into 0.1 M PBS containing 1% citrate for 2 h. The tissues were embedded, sliced, and stained with 2% uranyl acetate at room temperature for 15 min. The slices were air-dried overnight at room temperature, and then observed and photographed under an electron microscope (Thermo Fisher Scientific Co., Ltd., Waltham, MA, USA).

### Western blotting assay

The liver tissues were homogenized with lysate (RIPA:PMSF = 100:1) in an ice-water bath for 30 min. The homogenate was centrifuged at 4 °C and 12,000 rpm for 20 min, then the supernatant was taken, and the protein content was determined with a BCA protein quantification kit. Next, 5× loading buffer was added, and the proteins were denatured by boiling for 10 min. The amount of proteins in every well was 20 µg, and then the proteins were separated by electrophoresis (Bio-Rad, Hercules, CA, USA) through sodium dodecyl sulphate (SDS)-polyacrylamide gel electrophoresis (PAGE) (12% separation gel and 5% concentration gel) and transferred to nitrocellulose membranes. After blocking with 5% skim milk, the membranes were incubated with antibodies against Nrf2 (1:1000), HO-1 (1:400), β-actin (1:400), and β-tubulin (1:400) at 4 °C overnight, washed with TBST, and incubated with anti-rabbit (1:6000) and anti-mouse (1:8000) secondary antibodies for 1.5 h. Finally, an Ultra-sensitive Multi-function Imager (General Electric Co., Ltd., Boston, MA, USA) was used to expose the bands. The grey value was obtained by using ImageJ software to quantitatively analyse protein expression.

### Statistical analysis

All data were processed by using Statistical Package for the Social Sciences (SPSS) software program, version 23.0 (SPSS, Inc.). The data reported in this paper were presented as mean ± SD. One-way analysis of variance was used to compare multiple samples, and then Least-Square Difference (LSD) was performed for the *post hoc* test. *p* < 0.05 represented a significant difference.

## Results

### The UV fingerprint peaks of the three extracts were significantly different

**Figure 1.**
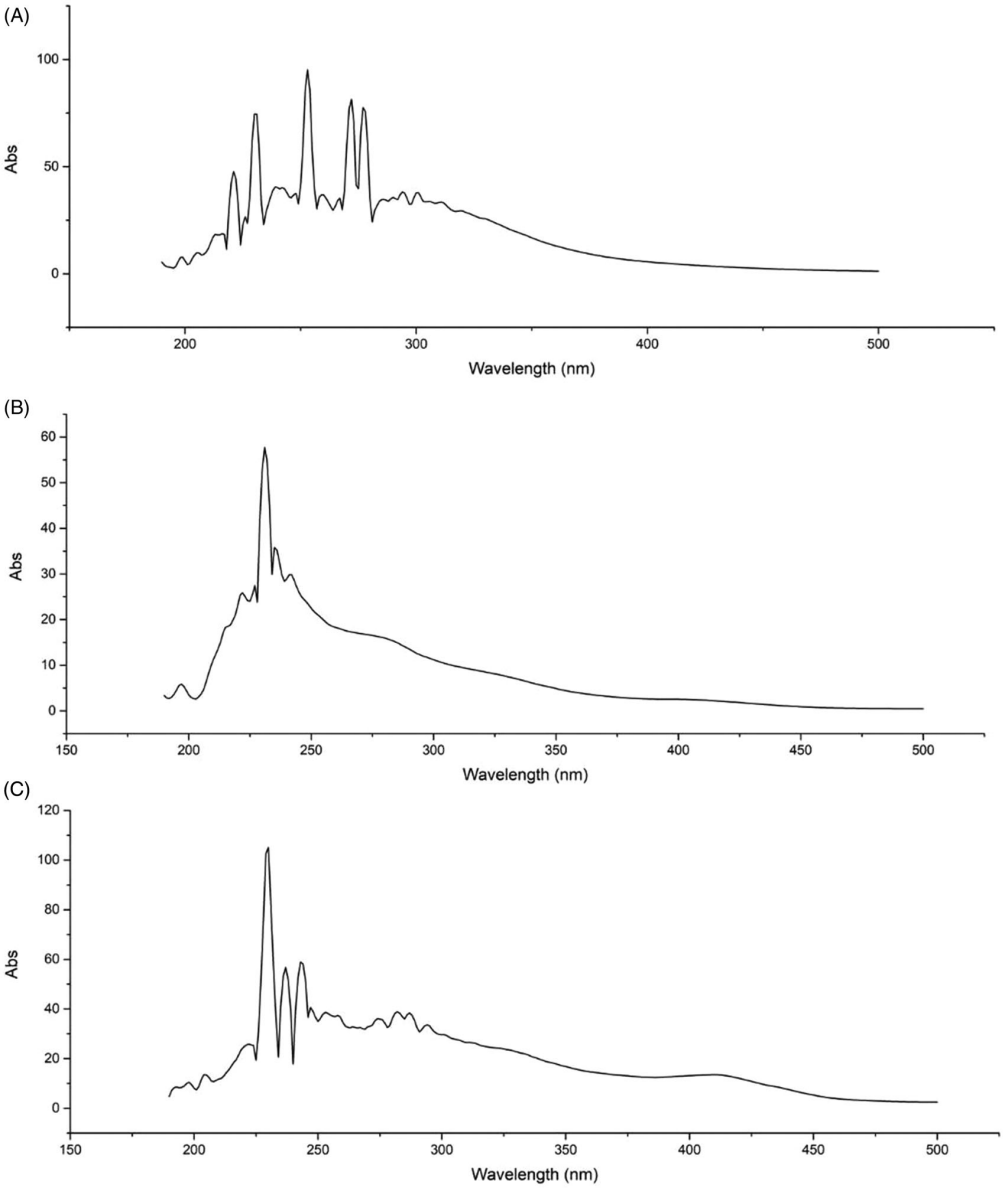
UV fingerprints of the ER, ES and EL of *C. serratus*. UV fingerprints of the ER (A), ES (B) and EL (C) of *C. serratus*. They had absorption peaks at the characteristic wavelengths.

As shown in the full-wavelength scanning spectra of the samples (Figure 1), the main absorption range was 200–300 nm. The ER had absorption peaks at 277, 272, 253, 230, and 221 nm; the ES had absorption peaks at 235, 231, and 227 nm; and the EL had absorption peaks at 243, 237, 230, and 222 nm. These finding illustrated that they had absorption peaks at characteristic wavelengths and no interference within the range of visible light.

### The HPLC fingerprint peaks of the three extracts were significantly different

**Figure 2.**
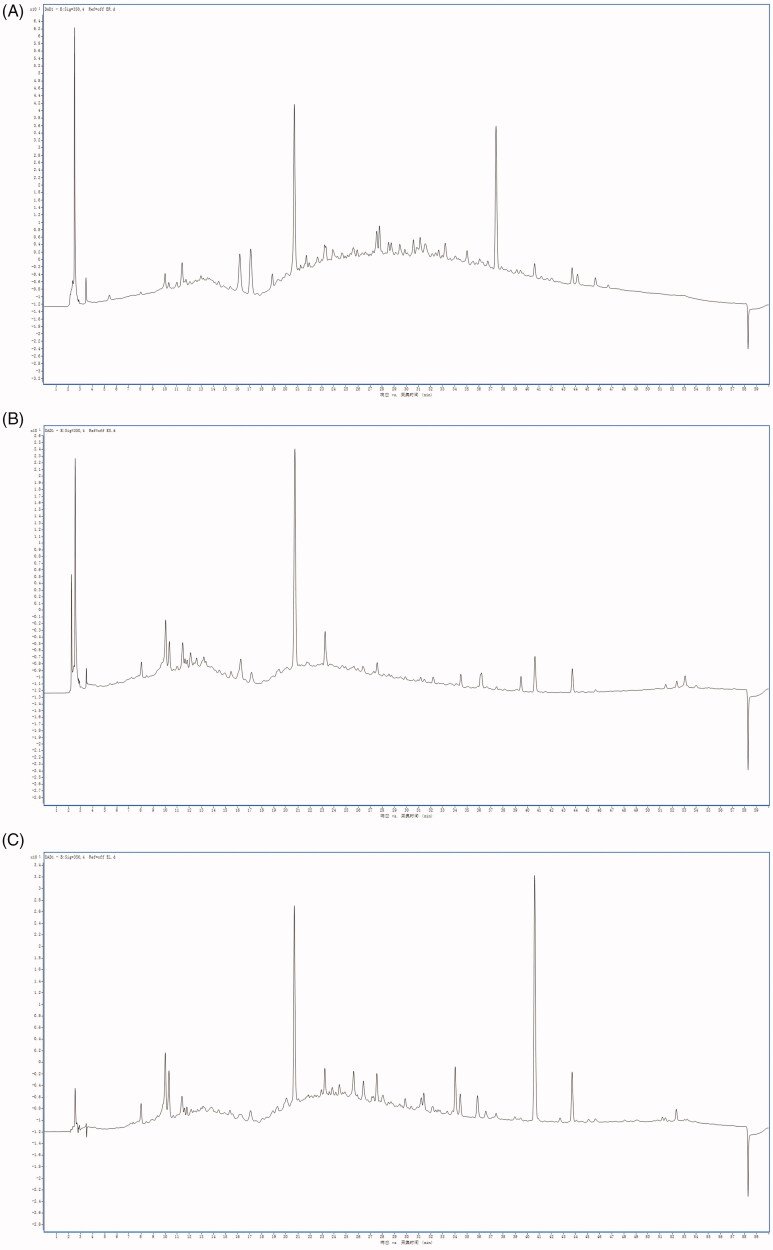
HPLC fingerprints of the ER, ES, and EL. HPLC fingerprints of the ER (A), ES (B), EL (C). They had absorption peaks at the characteristic retention times.

As seen from the HPLC scanning spectra of the samples (Figure 2), the main absorption peak range was 7.7–53.5 min. Thirteen major absorption peaks were obtained from the ER by HPLC with retention times of 10.0, 10.4, 16.2, 17.1, 20.8, 23.2, 33.5, 35.0, 37.2, 40.7, 43.8, 44.1, and 45.8 min, respectively. Eighteen main absorption peaks were obtained from the ES by HPLC with retention times of 8.0, 10.0, 10.2, 11.5, 12.1, 15.5, 16.2, 17.1, 20.8, 23.1, 27.5, 34.6, 36.1, 39.5, 40.7, 43.8, 52.2, and 53.0 min, respectively. Twenty-three main absorption peaks were obtained from the EL by HPLC with retention times of 8.0, 10.0, 10.2, 11.4, 17.1, 20.8, 23.2, 23.9, 24.5, 25.6, 26.5, 27.5, 28.0, 29.9, 31.2, 31.4, 34.0, 34.5, 35.7, 36.7, 40.6, 43.7, and 52.3 min, respectively. In other words, they had absorption peaks at the characteristic retention times, and there were no interferences.

### Optimization of experimental dosage by acute oral toxicity test

No death was observed when the oral doses of the ER, ES, and EL were 10.35, 8.05, and 2.90 g/kg, respectively. The LD_50_ of the ER, ES, and EL were found to be higher than 10.35, 8.05, and 2.90 g/kg, respectively. Therefore, it could be considered that the experimental doses (4.14 g/kg of the ER, 3.20 g/kg of the ES, and 1.16 g/kg of the EL) were less than the LD_50_. After pre-experiment, there were no death of a large number of animals at these doses, and the difference in toxicity of the three extracts could be detected at the same time. The doses of the three extracts were different due to different extraction rates. The amount of roots, stems, and leaves (raw medicinal materials) was identical, all of them were 3 × 0.018 × 5 × 100 = 27 (g). As a result, 4.14 g/kg of the ER, 3.20 g/kg of the ES, and 1.16 g/kg of the EL were selected as the doses for evaluating the hepatotoxicity of the three extracts.

### All extracts affected the general conditions of the rats in different degrees

The rats in the Con group had bright white fur, active reaction, normal intake and excretion. However, the rats in the ER group had slight red secretions in the canthus. And the rats in the ES group had slightly dull fur and red secretions in the mouth, nose, and canthus. The rats in the EL group had slightly yellow fur, and their activities were obviously reduced; secretions in the nose and canthus could be seen, and several rats had swollen toes and bleeding canthus.

### All extracts slightly changed the weight gain rates of the rats

**Figure 3.**
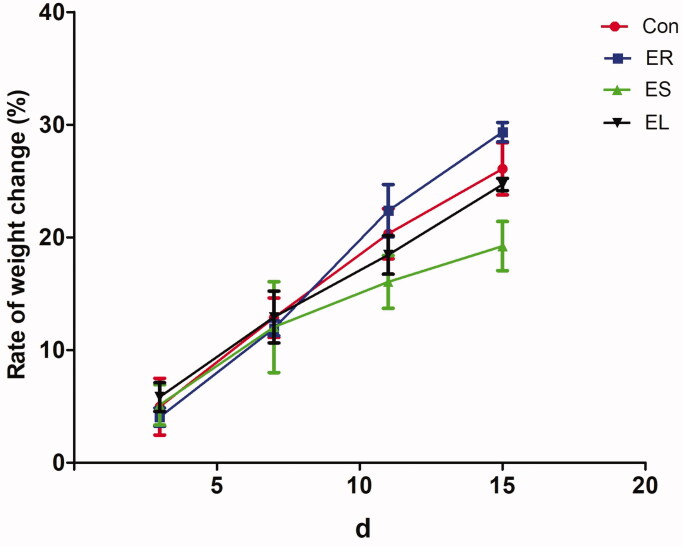
Changes in the body weights of the rats. The rats were treated with the ethanol extracts of different parts of *C. serratus* (ER: 4.14 g/kg/day, ES: 3.20 g/kg/day and EL: 1.16 g/kg/day) for 14 days. There were no significant changes in the body weights among the extract-treated groups. Data were presented as mean ± SD (n = 6).

The weight gain rates only slightly changed in all the extract groups compared with those in the Con group. Among them, the slight decreases in the weight gain rates in the EL and ES groups suggested that the EL and ES had damaging effects on the body weight, while there were no statistical significances. In contrast, the body weight gain rates of the rats in the ER group were slightly increased (Figure 3).

### All extracts had differential effects on the liver morphology

**Figure 4.**
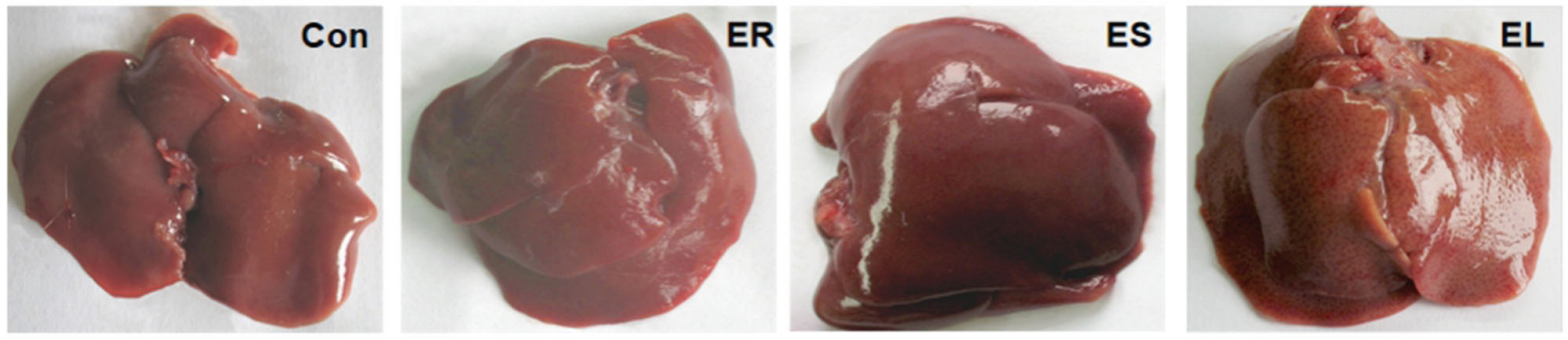
Effects on rat liver morphology. The rats were treated with the ethanol extracts of the different parts of *C. serratus* (ER: 4.14 g/kg/day, ES: 3.20 g/kg/day and EL: 1.16 g/kg/day) for 14 days. The Con group: normal; the ER group: dark red, smooth and regular edges; the ES group: low luster and hard texture; the EL group: rough, bruised, granular and hard texture.

In the Con group, the rat liver surface was smooth, glossy and normal in shape, bright red in colour and soft in texture. In the ER group, the rat liver was dark red with a smooth surface and regular edges, similar to those of the Con group. The rat liver in the ES group was dark red with irregular edges, loss of lustre and fibrosis. In the EL group, the rat liver was dark red with irregular edges, a rough surface, hard texture, and even bruises (Figure 4).

### All extracts increased the liver indexes in different degrees

**Figure 5.**
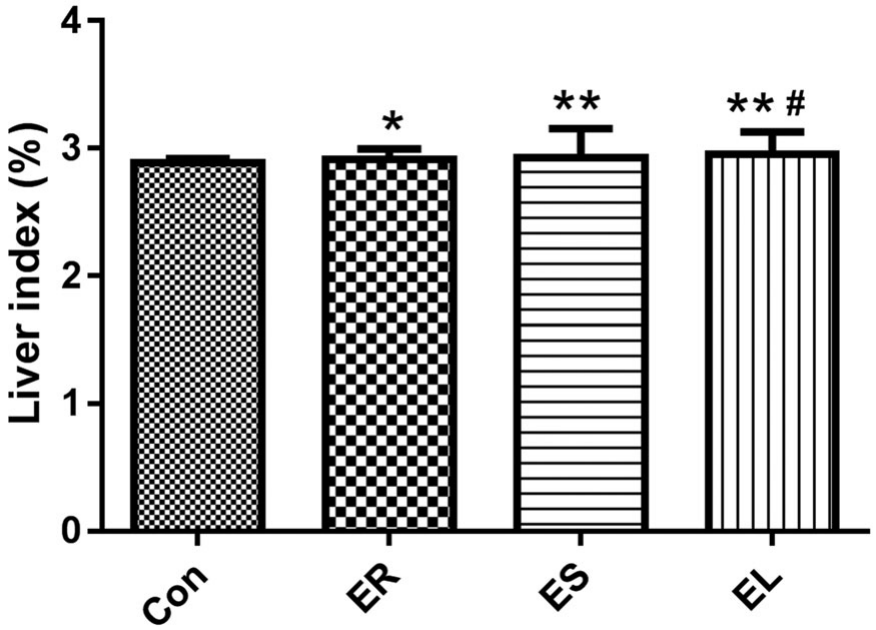
Effects on the liver index. The rats were treated with the ethanol extracts of the different parts of *C. serratus* (ER: 4.14 g/kg/day, ES: 3.20 g/kg/day and EL: 1.16 g/kg/day) for 14 days. The liver indexes were significantly increased in the extract-treated groups, and among them, it was highest in the EL group. Data were presented as mean ± SD (n = 6). *p < 0.05, **p < 0.01 vs. the Con group; ^#^*p* < 0.05 vs. the ES group.

As shown in Figure 5, the liver indexes of the rats exposed to the different ethanol extracts were obviously increased compared with that of the Con group (*p* < 0.05 or *p* < 0.01), especially in the ES and EL groups (*p* < 0.01). The liver index in the EL group was significantly higher than that in the ES group (*p* < 0.05).

### All extracts caused liver histopathological changes in different degrees

**Figure 6.**
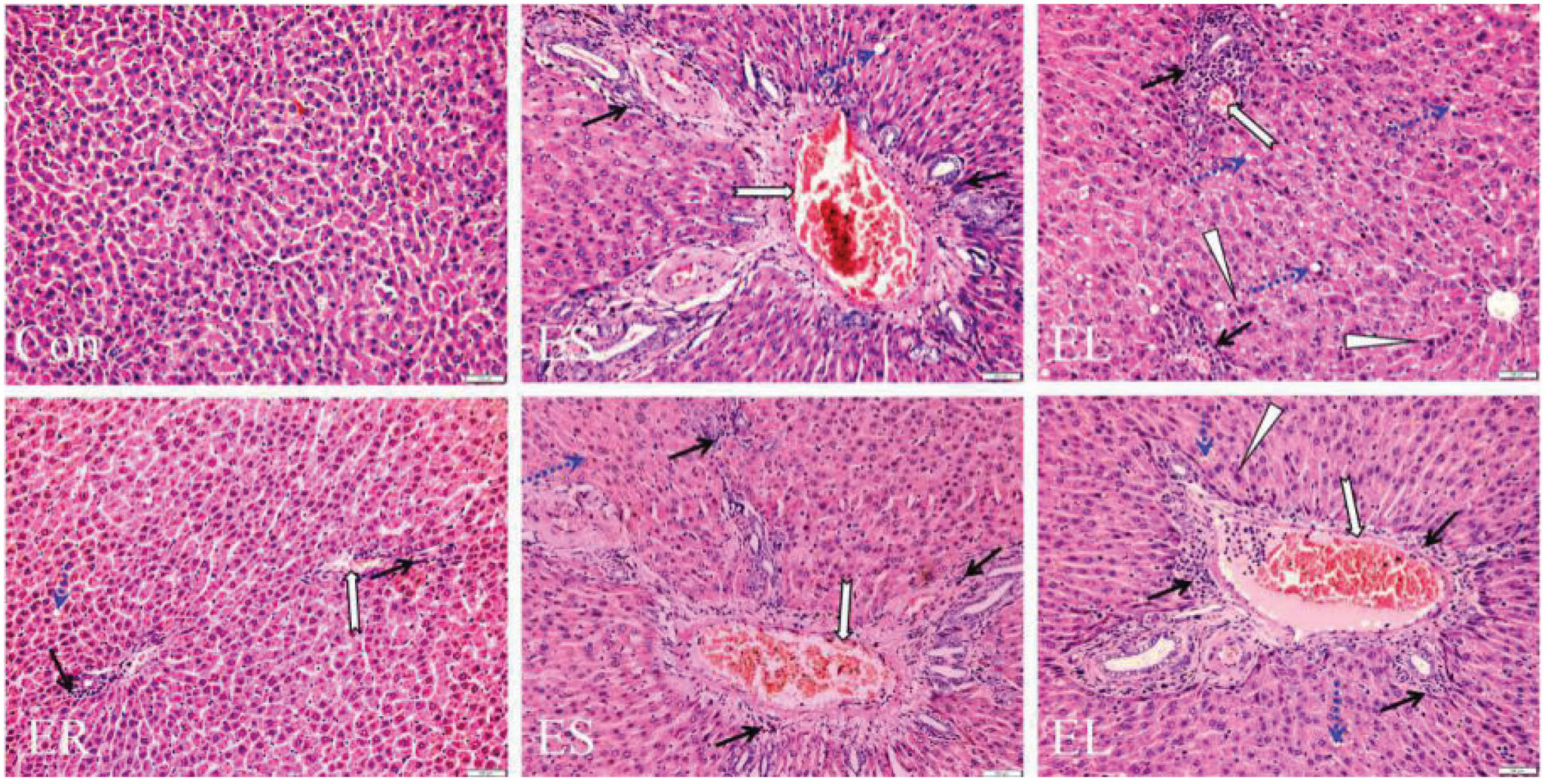
Representative microscopic photographs of the rat liver slices stained with H&E (200×). The rats were treated with the ethanol extracts of different parts of *C. serratus* (ER: 4.14 g/kg/day, ES: 3.20 g/kg/day and EL: 1.16 g/kg/day) for 14 days. **The Con group:** normal histological architecture and cell structure; **the ER group:** slight vasodilatation and congestion (
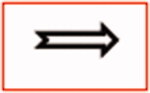
), inflammatory cell infiltration (
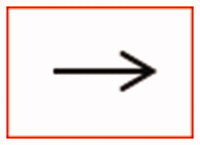
); **the ES group:** vasodilatation and congestion, inflammatory cell infiltration, steatosis (
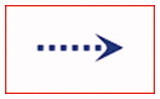
) and edema fluid; **the EL group:** vasodilatation and congestion, edema fluid, focal necrosis, eosinophilic degeneration (
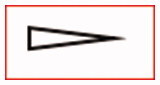
) and steatosis.

As shown in Figure 6, neatly arranged hepatic cells and clear hepatic lobules were seen in the livers of the Con group rats. The liver tissues of the extract group rats showed different degrees of damage. Among them, slight vascular congestion and inflammatory cell infiltration were observed in the ER group. The ES-treated rats showed vascular congestion with inflammatory cell infiltration, edoema fluid and steatosis. Administration of the EL caused a large amount of inflammatory cell infiltration, edoema fluid, focal necrosis, eosinophilic degeneration, and steatosis in the liver tissues. Unsurprisingly, the pathological scores of liver injury showed that the scores of the ES (7–11 points) and EL (15–18 points) groups were significantly higher than those of the Con group (0–1 point) (*p* < 0.01), and there were no significant changes in the scores of the ER group (2–3 points) compared with those of the Con group (Table 1).

**Table 1.** Liver histopathology scores of the rats in each group (mean ± SD, *n* = 6).

Group	Amount of administration (g/kg/day)	Liver histopathology score (points)
Con	/	0.73 ± 0.12
ER	4.14	2.70 ± 0.20^##^
ES	3.20	9.5 ± 1.76**
EL	1.16	16.37 ± 1.18^**,^^##^

**p < 0.01 *vs*. the Con group; ^##^*p* < 0.01 *vs*. the ES group.

### All extracts increased the positive expression of immunohistochemistry indicators in different degrees

**Figure 7.**
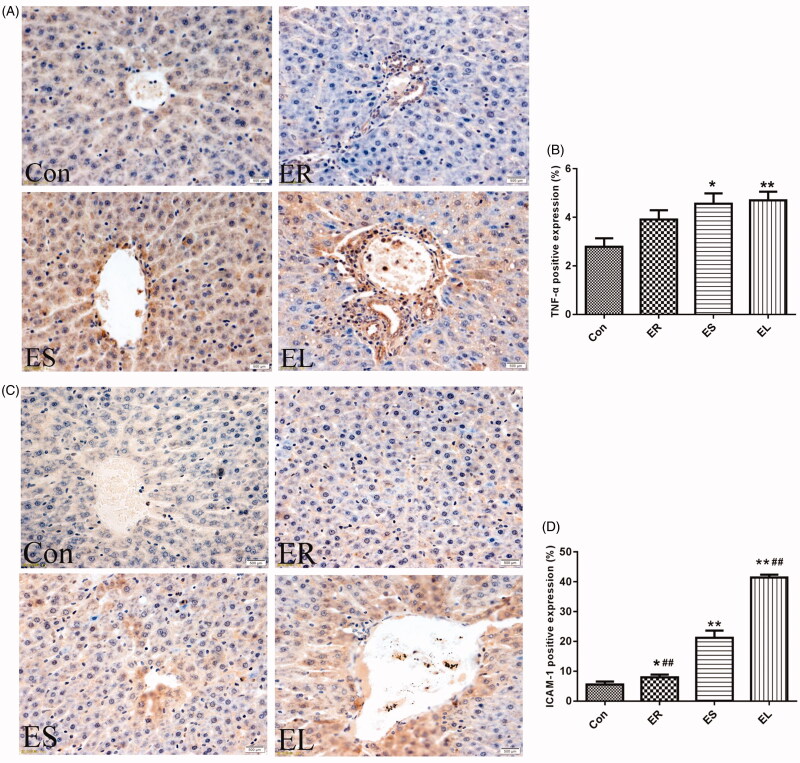
Expression of tumor necrosis factor-α (TNF-α) and intercellular cell adhesion molecule-1 (ICAM-1) in the liver tissues (400×). The rats were treated with the ethanol extracts of different parts of *C. serratus* (ER: 4.14 g/kg/day, ES: 3.20 g/kg/day and EL: 1.16 g/kg/day) for 14 days. (A) Immunohistochemistry results of TNF-α. The column chart represented the positive expression rate of TNF-α (B). (C) Immunohistochemistry results of ICAM-1. The column chart represented the positive expression rate of ICAM-1 (D). The positive expression of TNF-α in the ER group was not significantly different from that in the Con group, whereas its expression was significantly increased in the ES and EL groups. The expression of ICAM-1 in the treatment groups was significantly increased compared with that in the Con group. Data were presented as mean ± SD (n = 6). *p < 0.05, **p < 0.01 vs. the Con group.

TNF-α and ICAM-1 are mainly expressed and distributed diffusely in the cytoplasm, with a small number of brownish-yellow granules positively expressed on the cell membrane (Figure 7). Compared with the Con group, the positive expression of TNF-α in the ER group was not statistically significant, whereas its expression was significantly increased in the ES and EL groups (*p* < 0.05 or *p* < 0.01). The expression of ICAM-1 in the ER group was significantly increased (*p* < 0.05), whereas that in the ES and EL groups was dramatically increased (*p* < 0.01). Moreover, the expression of ICAM-1 in the EL group was significantly higher than that in the ES group (*p* < 0.01).

### All extracts changed the levels of liver biochemical indicators in different degrees

The effects of the different extracts of *C. serratus* on liver biochemical indicators were shown in Tables 2 and 3. Compared with the Con group, the extract groups had varyingly increased levels of TP, GLB, AST, ALT, ALP, and TBA, and decreased CHOL content. Moreover, for the changes of the above indicators, the EL group showed the most obvious differences, followed by the ES group.

**Table 2.** Serum biochemical indicators of the rats in each group (mean ± SD, *n* = 6).

Group	TP (g/L)	GLB (g/L)	AST (U/L)	ALT (U/L)
Con	59.03 ± 4.07	33.90 ± 3.27	132.00 ± 19.52	46.00 ± 26.00
ER	64.14 ± 5.75	40.70 ± 2.14**	167.80 ± 27.80	55.00 ± 15.95
ES	69.80 ± 4.76**	41.17 ± 2.77**	200.00 ± 35.00**	62.00 ± 17.58*
EL	71.12 ± 4.22**	42.30 ± 2.57**	247.34 ± 29.83**^#^	95.65 ± 16.43**^##^

TP: Total protein; GLB: globulin; AST: aspartate aminotransferase; ALT: alanine aminotransferase.

*p < 0.05, **p < 0.01 *vs*. the Con group; ^#^ *p* < 0.05, ^##^ *p* < 0.01 *vs*. the ES group.

**Table 3.** Serum biochemical indicators of the rats in each group (mean ± SD, *n* = 6).

Group	ALP (U/L)	TBA (µmol/mL)	CHOL (mmol/mL)
Con	128.00 ± 26.56	6.00 ± 1.00	1.58 ± 0.21
ER	143.20 ± 17.38	6.20 ± 1.84	1.47 ± 0.24
ES	149.67 ± 28.02	6.67 ± 1.58	1.24 ± 0.35*
EL	165.20 ± 32.64**	8.60 ± 1.14**^#^	1.18 ± 0.27**

ALP: Alkaline phosphatase; TBA: total bile acid; CHOL: cholesterol.

*p < 0.05, **p < 0.01 *vs*. the Con group; ^#^*p* < 0.05 *vs*. the ES group.

### All extracts changed the levels of liver oxidative stress indicators in different degrees

**Figure 8.**
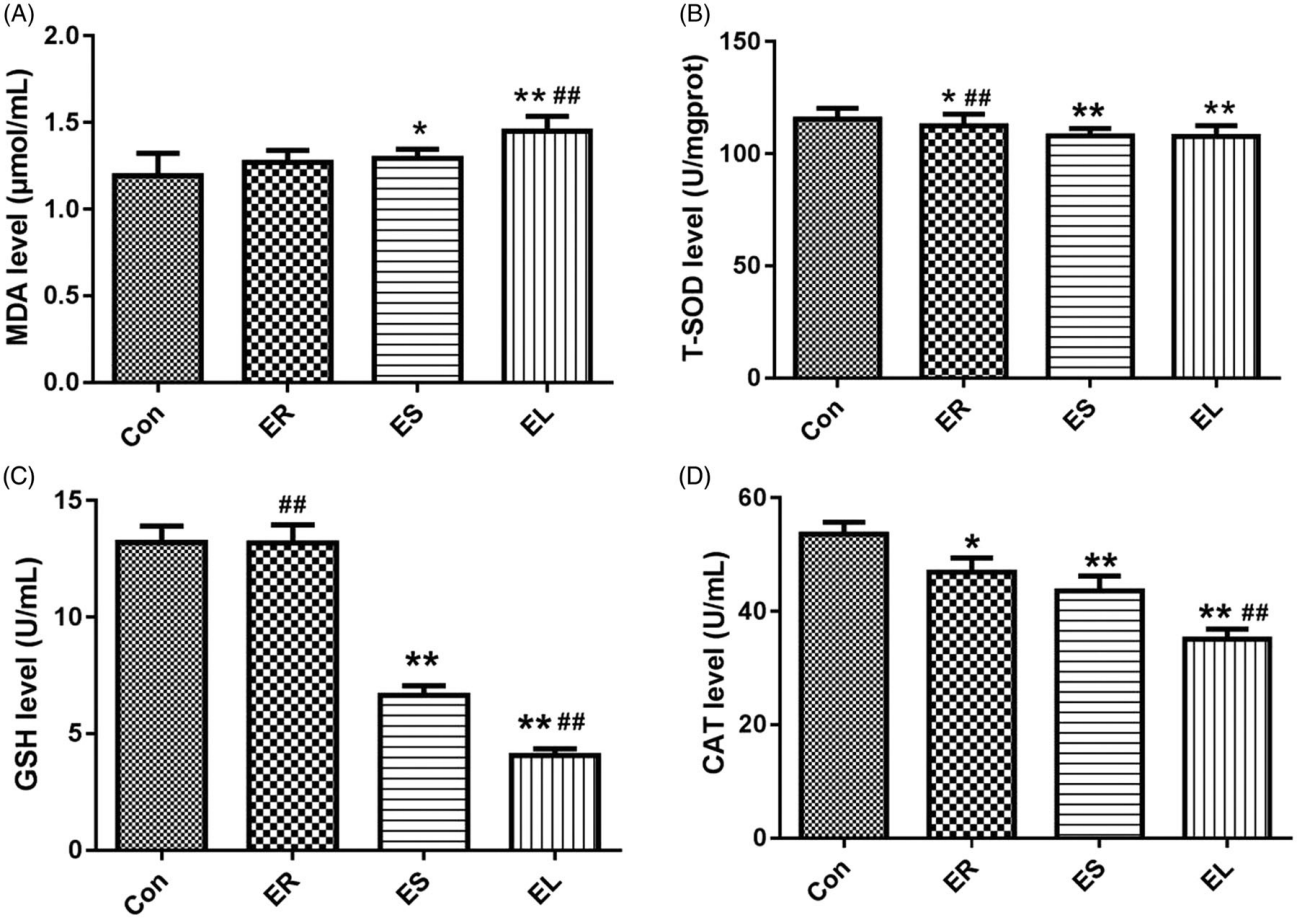
Effects on liver biochemical indicators. The rats were treated with the ethanol extracts of different parts of *C. serratus* (ER: 4.14 g/kg/day, ES: 3.20 g/kg/day and EL: 1.16 g/kg/day) for 14 days. The levels of malondialdehyde (MDA, A), total superoxide dismutase (T-SOD, B), glutathione (GSH, C) and catalase (CAT, D) differed considerably among the different groups. Data were presented as mean ± SD (n = 6). *p < 0.05, **p < 0.01 vs. the Con group; ^#^*p* < 0.05, ^##^*p* < 0.01 vs. the ES group.

The different extract-treated rats displayed changes in the levels of lipid peroxidation and antioxidant indicators, which were manifested as an increase in MDA content and decreases in T-SOD, GSH, and CAT contents as shown in Figure 8. Among them, the changes in the EL group were the most obvious, followed by the ES group.

### All extracts caused liver ultrastructural changes in different degrees

**Figure 9.**
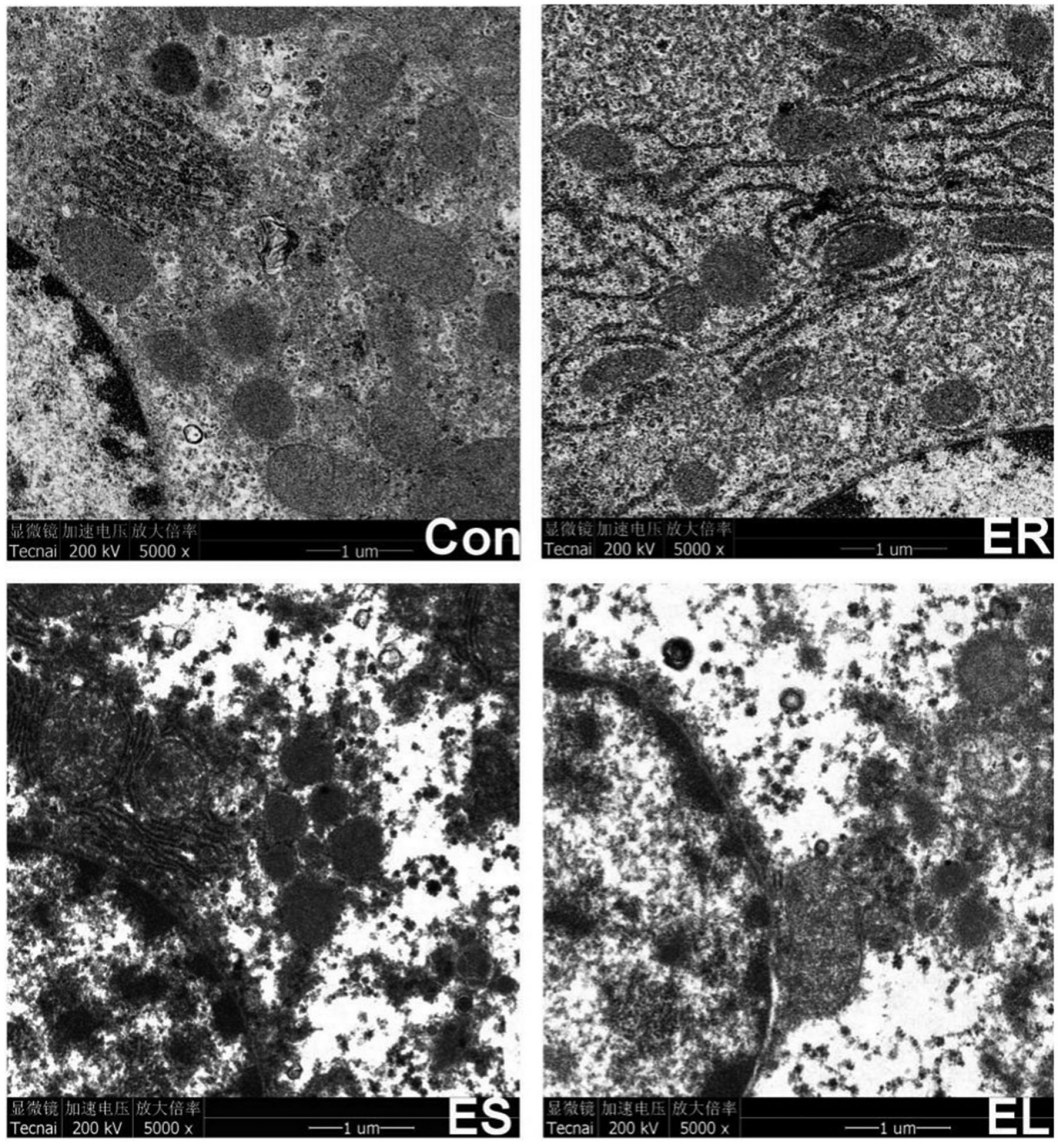
Electron micrograph of the liver tissues (5000×). The rats were treated with the ethanol extracts of different parts of *C. serratus* (ER: 4.14 g/kg/day, ES: 3.20 g/kg/day and EL: 1.16 g/kg/day) for 14 days. The Con group: normal structure; the ER group: mild expansion of the endoplasmic reticula; the ES group: mitochondrial structure deformation; the EL group: severely dilated, no intact structure.

Figure 9 showed that the ultrastructure (such as mitochondria and endoplasmic reticula) in the Con group was normal, while mild expansion of the endoplasmic reticula was observed in the ER group. Aggregation of chromatin, slight mitochondrial degradation, structural deformation, endoplasmic reticulum expansion and deformation, unclear boundaries, and a small number of vacuoles were observed in the ES group. Severely dilated endoplasmic reticula with deformed structure, uneven chromatin distribution, mitochondrial rupture, and a large number of vacuoles were observed in the EL group.

### All extracts inhibited Nrf2 and HO-1 protein expression in different degrees

**Figure 10.**
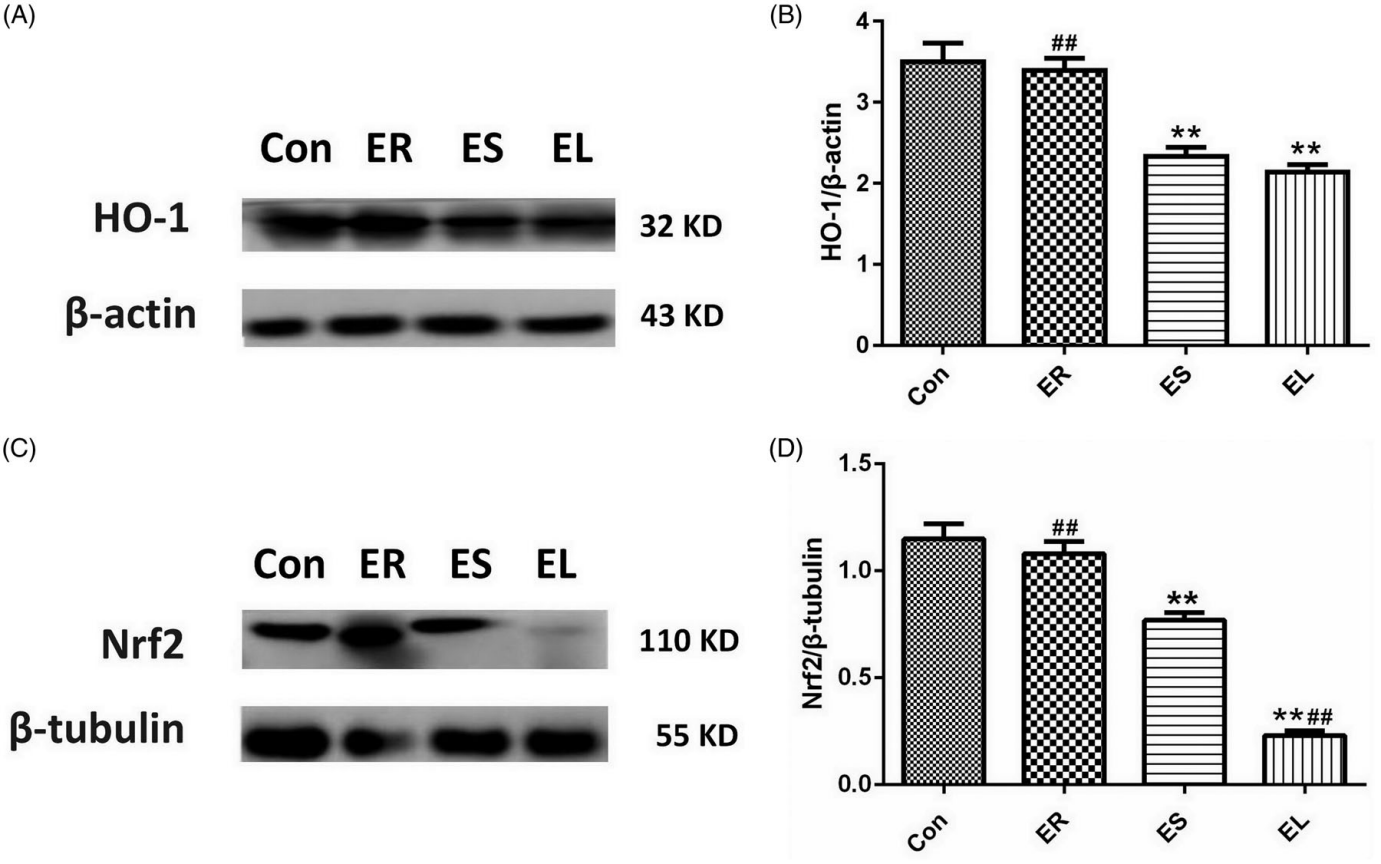
Expression of heme oxygenase-1 (HO-1) and nuclear factor erythroid-2 related factor 2 (Nrf2) proteins in the liver tissues. The rats were treated with the ethanol extracts of different parts of *C. serratus* (ER: 4.14 g/kg/day, ES: 3.20 g/kg/day and EL: 1.16 g/kg/day) for 14 days. (A, C) Western blotting results of the Nrf2/HO-1 pathway. Graphs represented the relative expression of HO-1 (B) and Nrf2 (D). Compared with that in the Con group, the HO-1 level in the ER group was slightly decreased, whereas it was significantly decreased in the ES and EL groups. The Nrf2 levels in the drug-treated groups were significantly decreased. Data were presented as mean ± SD (n = 6). *p < 0.05, **p < 0.01 vs. the Con group; ^#^*p* < 0.05, ^##^*p* < 0.01 vs. the ES group.

Figure 10 illustrated that the expression of HO-1 and Nrf2 proteins was slightly decreased (*p* > 0.05) in the ER group compared with that in the Con group. In contrast, the expression levels of Nrf2 and HO-1 in the ES and EL groups were significantly decreased (*p* < 0.01). The reduction of Nrf2 protein expression in the EL group differed significantly from that in the ES group (*p* < 0.01).

## Discussion

The toxicity of TCM is often ignored in extensive clinical practice. In order to make effectively use of TCM, the standard of usage and the dosage of TCM need to be further improved. Moreover, it is noteworthy that the relationship between the effective parts and the toxic parts of some poisonous medicinal plants remains to be studied (Teschke et al. 2013). Its toxicity and medical effect should be comprehensively considered in the clinical application of TCM. In recent years, the researches on *C. serratus* have mainly focussed on the analysis of its components and pharmacological effects, however, its hepatotoxicity was largely neglected.

The pathological changes in rat liver reflect the degree of liver damage. Based on our findings, the ER led to slight vascular congestion and inflammatory cell infiltration; the ES and EL caused local necrosis, obvious inflammatory cell infiltration, vascular congestion, edoema fluid and steatosis, which revealed that the ES and EL had much stronger hepatotoxicity in rats.

The decreases in the weight gain rates of the rats in the ES and EL groups indicated the emergence of toxicity. The rate of weight gain of the ER group was higher than that of the Con group, and this effect might be related to the nutrient-rich, low-toxicity, and anti-inflammatory effects of *C. serratus*. The liver index was increased significantly in the *C. serratus* extract-treated groups, especially in the ES and EL groups. Therefore, the toxicity of the EL and ES was much higher than that of the ER. These findings were consistent with the pathological results of the liver.

TNF-α, a proinflammatory factor, worsens damage to hepatocytes, leads to inflammation under excessive conditions, and plays an important regulatory role in inflammatory reactions (Suliman et al. 2012). The positive expression of TNF-α reflects the damage degree to hepatocytes (Li et al. 2015). Moreover, TNF-α also causes significant increases in the levels of ALT and AST (Malik et al. 2019), and induces the expression of ICAM-1 via downregulation of the Nrf2/HO-1 signalling pathway (Chen et al. 2020). ICAM-1 is recognized as an adhesion molecule associated with specific immunity and inflammatory effects, which mediates neutrophils to pass through the whole layer of endothelial cells. Then, neutrophils migrate to hepatocytes to release proteases and reactive oxygen species (ROS), causing hepatocyte damage (Woolbright et al. 2014). Under normal circumstances, ICAM-1 is expressed at low level, whereas hepatotoxic drugs may increase its expression, further aggravating the inflammatory reaction (Zhou and Sui 2019). In this study, the positive expression rates of TNF-α and ICAM-1 were both increased after exposure to the *C. serratus* extracts. In addition, the effects of the EL were stronger than those of the ES. This toxicity result could also be observed at the histological level.

ALT, AST, and ALP are markers of hepatocyte injury, and their elevation in serum reveals liver damage (Attia et al. 2016). ALT is regarded as the ‘gold standard’ for the clinical biochemical detection of liver injury due to its sensitivity, and 1% of hepatocyte necrosis can double the ALT level (Heydrnejad et al. 2015). AST mainly exists in the mitochondria of hepatocytes and is released into the blood when mitochondria disintegrate due to injury and rupture of hepatocytes (Ennulat et al. 2010). ALP is one of the main markers of hepatobiliary function and cholestasis; in addition, an elevated ALP level indicates adverse effects of drugs on liver function (Ma et al. 2018).

TBA is the only indicator that simultaneously reflects metabolism, secretion status, and cell damage in the liver (Lemoinne et al. 2018). It is rarely present in serum but significantly increased in acute hepatitis (Xu et al. 2012; Ai et al. 2013). In response to hepatocyte injury, TBA promotes the rate-limiting enzyme activity of CHOL synthesis, resulting in a decrease in CHOL content (El-Hawary et al. 2019). TP is composed of albumin (ALB) and GLB. ALB and GLB will increase in response to chronic liver disease or other liver damage, as does the TP (Silva-Carvalho et al. 2019), and a significant increase in the level of TP induces oxidative stress. In this study, the significant increases in the TP contents in the ES and EL groups suggested hepatotoxicity to a certain degree. The hepatotoxicity in the ES group was demonstrated by significant increases in AST, ALP, GLB, and TP levels. Moreover, the level of CHOL was markedly decreased, whereas other indicators were obviously increased in the EL group. Only GLB was significantly increased in the ER group. The above results indicated that both the ES and EL had severe hepatotoxicity in rats.

Oxidative stress injury is the main mechanism of liver injury, reflecting the imbalance between free radicals and antioxidants (Hasanein et al. 2016). MDA, SOD, GSH, and CAT are indicators closely related to the mechanism of hepatotoxic oxidative stress injury (Wu and Cederbaum 2009). MDA, a cytotoxic substance, can be used to assess the levels of oxygen free radicals and the severity of tissue oxidative stress damage (El-Agamy et al. 2016). Among them, SOD is an important antioxidant enzyme, which can scavenge oxygen free radicals in the body and protect the body from tissue damage mediated by oxidative stress (El-Agamy et al. 2016). CAT can decompose H_2_O_2_ and terminate free radical chain reaction. Its level can measure the body’s ability to resist oxidative stress damage (Xu et al. 2017). GSH promotes the antioxidant defense system of the liver by scavenging hydroxyl free radicals and superoxide free radicals (Yin et al. 2019). This study revealed that the EL group showed an obvious elevation in MDA content and significant reductions in SOD, CAT, and GSH levels, thus clarifying the significant reductions of free radical scavenging and antioxidant capacity, leading to a large quantity of free radical production, indicating severe hepatotoxicity. Whereas the lower MDA level and higher SOD, CAT, and GSH levels in the ER group indicated that hepatotoxicity was minimal.

This study also revealed that all extracts of *C. serratus* changed the ultrastructure of the liver to varying degrees, such as dilation and degranulation of the rough endoplasmic reticula and mitochondrial swelling and vacuolation, which were closely related to hepatotoxicity. Lipid deposition was observed in the cytoplasm of the EL and ES group rats, causing hepatic steatosis; an increase in the level of MDA is closely related to lipid peroxidation, which was reflected in the ultrastructural damage of mitochondrial membranes.

The Nrf2/HO-1 signalling pathway is considered as an important endogenous antioxidant signalling pathway. Nrf2, a key nuclear transcription factor, is mainly present in the cytoplasm. It enters the nucleus in response to oxidative stress and promotes the expression of protective indicators (SOD, GSH, CAT, HO-1, etc.) to resist external damage (Jeong et al. 2006; Wang et al. 2018). The expression of the Nrf2 protein may be inhibited in cases of body injuries (Loboda et al. 2016).

As a downstream molecule regulated by Nrf2, HO-1 plays an important role in protecting organisms from oxidative damage (Jeong et al. 2019). The expression level of the HO-1 protein may decrease after prolonged toxic stimulation (Shi et al. 2018). In this study, compared with the Con group, the HO-1 level in the ER group was slightly decreased, whereas it was significantly decreased in the ES and EL groups. The Nrf2 levels in all the drug groups were significantly decreased, especially in the ES and EL groups, which revealed that the liver injuries of the ES and EL groups were much more severe than that of the ER group.

Previous studies had shown that among the ethanol extracts of the roots, stems, and leaves of *C. serratus*, the ethanol extract of the roots had the least cardiac renal toxicity (Sun et al. 2019a, 2020a). What’s more, the ethanol extracts of the roots, stems, and leaves of *C. serratus* could alleviate inflammation in adjuvant arthritis rats, and the root extract had the best anti-inflammatory activity (Sun et al. 2020b). Moreover, among the chloroform, ethyl acetate, *n*-butanol, and water separated parts of the root extract, the water separated part exerted the best anti-inflammatory activity in a dose-dependent manner in LPS-stimulated macrophages (Sun et al. 2019b). The results of this study combined with previous studies showed that the application of *C. serratus* root is expected to become an effective clinical treatment of inflammatory diseases.

## Conclusions

Both the ES and EL of *C. serratus* induce liver injury in rats, and the ER has the least hepatotoxicity, which may be related to oxidative stress and suppression of the Nrf2/HO-1 pathway. It shows that the root extract is the least toxic and the most effective, which illustrates that the root is a better medicinal part.

In the near future, we will determine the levels of direct bilirubin, indirect bilirubin and Nrf2 phosphorylation in female rats to further reveal the toxicity and anti-inflammatory activity of the root extract of *C. serratus* so as to provide guidance for its clinical safe applications.

## Data Availability

All the data generated and analysed during this study are included in this published article.
